# Dietary Intake of Recreational Endurance Runners Associated with Race Distance—Results from the NURMI Study (Step 2)

**DOI:** 10.3390/nu14183698

**Published:** 2022-09-07

**Authors:** Derrick Tanous, Karl-Heinz Wagner, Claus Leitzmann, Mohamad Motevalli, Gerold Wirnitzer, Thomas Rosemann, Beat Knechtle, Katharina Wirnitzer

**Affiliations:** 1 Department of Sport Science, Leopold-Franzens University of Innsbruck, 6020 Innsbruck, Austria; 2 Department of Research and Development in Teacher Education, University College of Teacher Education Tyrol, 6010 Innsbruck, Austria; 3 Department of Nutritional Sciences, University of Vienna, 1090 Vienna, Austria; 4 Institute of Nutrition, University of Gießen, 35390 Gießen, Germany; 5 AdventureV & Change2V, 6135 Stans, Austria; 6 Institute of Primary Care, University of Zurich, 8091 Zurich, Switzerland; 7 Medbase St. Gallen Am Vadianplatz, 9001 St. Gallen, Switzerland; 8 Research Center Medical Humanities, Leopold-Franzens University of Innsbruck, 6020 Innsbruck, Austria

**Keywords:** nutrition, diet, food frequency, running, half-marathon, marathon, ultra-marathon, endurance, physical activity, health

## Abstract

While the popularity of distance running is growing worldwide, endurance runners’ dietary challenges associated with their prolonged training and racing activities have not yet been fully understood. The present investigation was conducted with the aim of examining the association between race distance and dietary intake of distance runners. A total of 317 runners initially participated, and after data clearance, 211 endurance runners (57% females) were finally considered the study sample. Runners were assigned to three race distance groups: 10-km (*n* = 74), half-marathon (*n* = 83), and marathon/ultra-marathon (*n* = 54). An online survey was used to collect data; dietary intake was monitored using a comprehensive food frequency questionnaire, including 53 food groups categorized in 14 basic and three umbrella clusters. There was no significant difference (*p* > 0.05) between race distance groups in consumption of most food clusters except for “fruits and vegetables” and “total of protein”, with a predominance of 10-km runners compared to half-marathoners and (ultra-)marathoners (*p* ≤ 0.05). Age was a significant predictor for the consumption of only five (out of 17) food clusters (*p* ≤ 0.05), including “fruit and vegetables”, “unprocessed meat”, “processed meat”, “eggs”, and “plant protein”. Future investigations with a larger sample size and more differentiated (sub)groups may help provide comparable data to develop a better understanding of the dietary behaviors among shorter versus longer distance runners.

## 1. Introduction

Distance running is a low- to moderate-intensity prolonged physical activity performed over 10-km to ultra-marathon runs [1]. During the past decade, a remarkable increase has been reported in distance running popularity, with a further boom from the commencement of the COVID-19 pandemic [1,2]. Distance running is performed with different motives, such as health promotion, pleasure, weight control, personal achievement, and social reasons [3,4].

Nutritional strategies and dietary patterns may predict the nutrient requirements of athletes and play significant roles in the adaptations and performance of endurance runners [5,6]. While the consumption of high-carbohydrate meals has been recommended to endurance runners on pre-event days and hours within carbohydrate loading strategies as part tapering strategies, they are advised to avoid high-fat, high-protein, and high-fiber foods during pre-competition hours [5,7]. Regardless of in-race nutritional tactics, however, there are predominant challenges about the daily nutritional requirements of endurance runners to support their training adaptations and health status [8]. Nutritional strategies of distance runners should not only support the higher rate of energy expenditure but are also crucial to cover the greater exercise-induced thermoregulatory demands and the increased resting metabolic rate of endurance runners compared to general populations [9,10].

Compared to general populations, endurance athletes are reported to have a higher frequency, diversity, and quantity of food intake [9,11]. Despite this fact, it has been documented that both recreational and elite endurance athletes seem to be at risk of sparse bioavailability of energy [12,13], which can be caused by poor dietary patterns [14]. While the increased duration and frequency of training/running sessions can expand the risk of caloric undersupply [7,10], this caloric imbalance can deteriorate further when accompanied by sport-specific challenges, such as difficulty matching meal frequencies with training and/or gastrointestinal distress, which are prevalent among athletes [15,16]. Inadequate or imbalanced dietary intake may lead to clinical and non-clinical nutrient deficiencies in endurance athletes that could result in adverse health effects and unfavorable consequences for performance, including muscle catabolism, diminished bone mineral density, increased risk of injury, immune function abnormalities, and more [17,18]. 

Regardless of daily nutritional requirements, endurance runners (especially those who run over longer distances) are reported to encounter a high level of physical stress during training and racing activities that could be associated with muscle damage, soreness, and/or inflammation [7,19]. As is recommended by the International Society of Sport Nutrition (ISSN), nutritional strategy (particularly sufficient consumption of carbohydrate, protein, and metabolically needed micronutrients) plays an important role in the management of the physical pressure and acceleration of post-running recovery [7,20]. However, it seems that there is a difference between various groups of endurance athletes considering nutritional patterns. Evidence shows that training in a fasting state is more prevalent in ultra-endurance runners than in those who run over shorter distances [21]. It has also been reported that marathoners and ultramarathoners have a greater tendency to follow the associated nutritional recommendations compared to shorter-distance runners [22]. In addition, despite the improved health-related behaviors regarding food choice among all groups of distance runners, half-marathoners are reported to have a general tendency towards a better health status compared to other distance runners [4]. These data provide initial evidence to suggest the necessity to apply specified nutritional examinations and recommendations based on different groups of distance runners.

Assessment of dietary intake is a key part of sports nutrition practice, enabling specialists to detect nutritional undersupply and deficiencies and consequently optimize health- and performance-related nutritional strategies. A well-examined dietary assessment can help to develop individualized nutrition plans helping athletes follow general dietary guidelines. Despite the differences in health and nutritional challenges/concerns of shorter vs. longer distance runners, to date, scientific studies investigating dietary intake and patterns of distance runners [15,23,24,25] are neither adequate to conclude solid dietary recommendations nor distinguish runners over different race distances. This fact supports the general caution that is advised when comparing and interpreting nutritional behaviors of endurance runners [26]. Given the increasing popularity of distance running over varied distances and considering the importance of nutrition in endurance runner success, it appears crucial to examine and compare the dietary intake of endurance runners across different race distance subgroups to provide practical knowledge for expanding personalized nutritional strategies. Thus, the present study aimed to examine the dietary intake of endurance athletes running over 10 km, half-marathon, and (ultra-)marathon race distances to identify potential distance-related differences.

## 2. Materials and Methods

### 2.1. Study Design and Ethical Approval

This study is a part of the Nutrition and Running High Mileage (NURMI) Study Step 2. The protocol of the study was approved by the ethical board of St. Gallen, Switzerland (EKSG 14/145; 6 May 2015) with the trial registration number ISRCTN73074080 [27]. Detailed information about the methods of the NURMI Study Step 2 has been previously presented elsewhere [27,28].

### 2.2. Participants and Experimental Approach

Endurance runners were mostly from German-speaking countries (i.e., Germany, Austria, and Switzerland). They were contacted and invited to participate in different ways, including social media, running communities, email lists of running magazines, websites of organizers for marathon events, and other multi-channel recruitments. Runners were requested to complete the online survey of the NURMI Study Step 2, available in English and German (at https://www.nurmi-study.com/en; accessed on 20 July 2022). After providing a written description concerning the study objectives and procedures, participants provided informed consent before filling in the questionnaire. Four initial inclusion criteria were compulsory for successful participation in the present study: (1) written informed consent; (2) at least 18 years of age; (3) questionnaire Step 2 completed; and (4) successful participation in an ultra-endurance running event (at least half-marathon) in the past two years.

Initially, participants were differentiated based on race distance and classified as half-marathoners and (ultra-)marathoners. For the latter group, data were combined since the marathon distance is covered within an ultra-marathon. The shortest distance reported for ultra-marathon was 50 km, while the longest distance was 160 km. However, 74 runners reported completing the 10-km distance but had not successfully participated in a half-marathon. As they had provided accurate data, and in order to avoid losing these valuable data sets, participants who met the inclusion criteria 1-3 were kept as another race distance group, i.e., 10-km runners. Therefore, 10-km, half-marathon, and marathon/ultra-marathon were defined as study groups. In addition, participants were categorized according to their self-reported diet types: omnivores (those with no restriction on any food items); vegetarians (those who avoid all flesh foods, including fish and shellfish, but consume egg and/or dairy); and vegans (those who avoid any type of food from animal sources) [29,30].

### 2.3. Data Clearance

**Figure 1:**
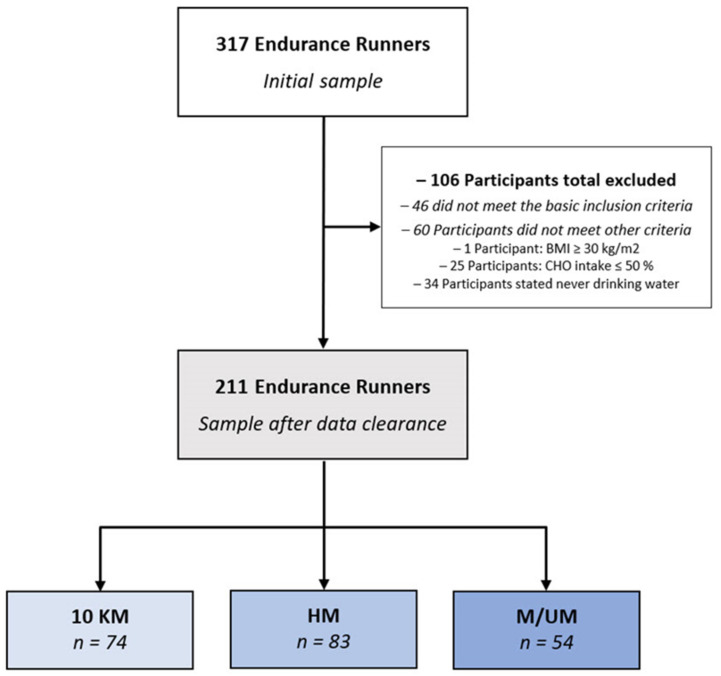
Participants’ enrollment and classifications by race distance. BMI—body mass index. CHO—charbohydrates. 10 KM—10-kilometers. HM—half-marathon. M/UM—marathon/ultra-marathon.

The study’s initial sample included 317 runners and 106 of whom were excluded from data analysis. Of the excluded participants, 46 participants were not able to meet the basic inclusion criteria. Based on the World Health Organization (WHO) standards [31,32], the body mass index (BMI) approach was implemented in order to control for a minimal health status associated with a minimum fitness level, thus further enhancing the reliability of data sets. As a result, participants with a BMI ≥ 30 kg/m^2^ were excluded from the study due to the higher priority of health-protective strategies compared to running, which could have contradicted the findings. In addition, 25 runners who were observed to have a diet with ≤50% carbohydrates (which is lower than the lowest level recommended for maintaining health-performance association) were further excluded [6,33,34]. Furthermore, 34 participants with implausible reports regarding water intake (e.g., never drinking water) were also excluded from the examination in order to avoid conflicting data [33]. The final sample for statistical analysis included 211 runners with complete data sets. Figure 1 displays the enrollment and classification of participants.

### 2.4. Measures and Analytical Modelling

The validated food frequency questionnaire (FFQ) from the “German Health Interview and Examination Survey for Adults (DEGS)” (permitted by the Robert Koch Institute, Berlin, Germany) [35,36] was used for the present investigation. Endurance runners were asked to report their regular food intake in the past four weeks based on the consumption frequency (single-choice out of 11 options ranging from “never” to “5 times a day”) and quantity of wide-ranging specific dietary items (single-choice with several options), including meals/foods eaten while out such as restaurants, canteens, meetings, and parties.

There were 53 food groups based on the DEGS-FFQ and using the Nova classification system developed by the Food and Agriculture Organization (FAO) of the United Nations [37,38,39,40], food groups were classified into 17 food clusters for advanced quantitative and qualitative data analysis (Table 1). Self-reported information was linked to race distance groups and included sociodemographic data, general motives for following a specific kind of diet, and food frequency reports.

### 2.5. Statistical Analysis

The statistical software R version 4.1.1 (R Foundation for Statistical Computing, Vienna, Austria) was used for all statistical analyses. Descriptive statistics were conducted for exploratory analysis with mean values and standard deviation (SD) and median and interquartile range (IQR). To examine the differences between diet type groups, univariate tests were used. Chi-square tests (χ^2^; nominal scale) were performed to test the association between race distance and sex, academic qualification, nationality, marital status, diet type, and the associated motives. Kruskal–Wallis test (ordinal and metric scale; F distributions or ordinary least squares, standard errors (SE), and R^2^) was done to test the association of race distance with body weight, height, BMI, and age.

As the latent variable, food clustering was derived using 53 manifest parameters (assessing amount and frequency of consumption of specific dietary items). To scale the dietary intake presented by measures, items, and clusters, a heuristic index (as a new compound variable) ranging between 0 and 100 was developed for all items, and FFQ was computed by multiplying the two questions and dividing by the maximum. 

Linear regression was conducted to examine differences in consumption of specific food clusters by race distance and age. Effect plots with the standardized regression coefficient (*β;* standardization using z-score) and 95% confidence interval (95%-CI) were designed to show the differences in respective food clusters between race distance groups. The statistical significance level was set at *p* ≤ 0.05.

## 3. Results

Totally, 211 runners with a median age of 38 (IQR 18) years were included in statistical analysis. Based on race distances, there were 74 10-km runners (74.3% females), 83 half marathoners (55.4% females), and 54 (ultra-)marathoners (37.0% females). Endurance runners were mostly (96%) from German-speaking countries (Germany, Austria, and Switzerland; also known as D-A-CH countries). Descriptive data indicated that there were significant differences between the runners of different distances in weight (*p* = 0.003) and height (*p* = 0.004) but not BMI (*p* > 0.05). A significant sex difference was found between race distance groups (*p* < 0.001), where the distribution of females was higher in 10-km (74% vs. 26%) and half-marathon (55% vs. 45%) runners and lower in marathon/ultramarathon (37% vs. 63%) runners. There was no significant difference (*p* > 0.05) between 10-km, half-marathon, and (ultra-)marathon runners in age, academic qualification, nationality, marital status, and diet type. Among the motives that runners reported to justify their adherence to the self-reported diet types, “health & well-being” was the only motive with a significant between-group difference (*p* = 0.024). Table 2 shows the sociodemographic information of the runners over 10-km, half-marathon, and (ultra-)marathon distances.

The analysis of food frequency data showed significant differences between race distance groups in consumption of two food clusters, including “fruit and vegetables” (*p* = 0.010) and “protein” (*p* = 0.016), where 10-km runners had a higher intake of both food clusters compared to HM and M/UM runners. However, among the food items under the subset of the “fruit and vegetables” cluster, there was a significant predominance of 10-km runners only in vegetables (*p* = 0.012) but not for fruit (*p* = 0.689) or vegetable juice (*p* = 0.440). No significant differences were found between male and female runners in consumption of the remaining 15 food clusters or their subset food items (*p* > 0.05). Table 3 displays the differences between 10-km, HM, and M/UM runners in their self-reported consumption of food cluster and the subset items within the past 4 weeks.

**Figure 2:**
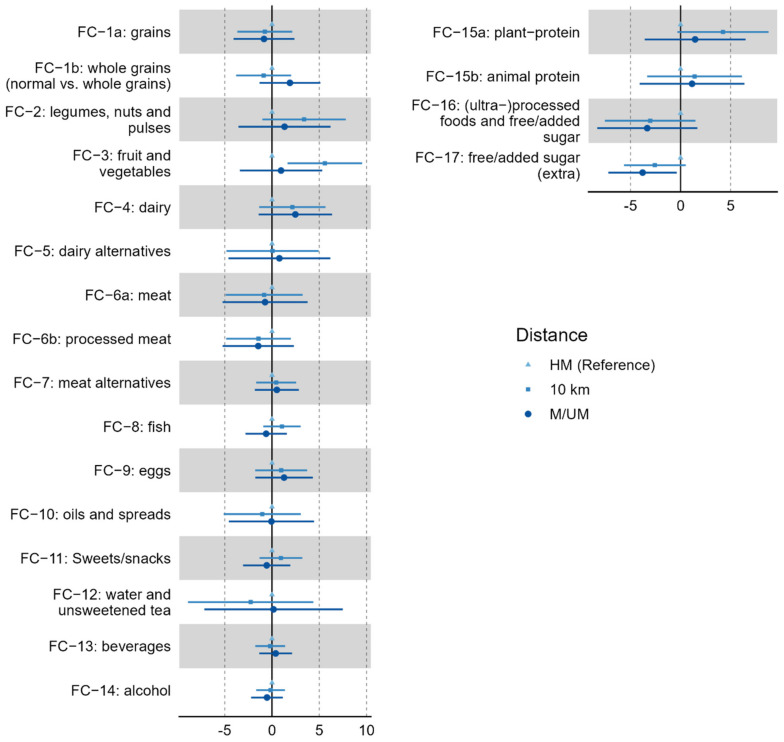
Forest plots with standardized regression coefficient, including 95% of confidence interval to display differences between race distance groups in basic (left graphic) and umbrella (right graphic) food clusters. The half-marathon group is considered the reference, and the differences are shown based on variations of 10-km and (ultra-)marathon runners from half-marathoners. FC—food clusters. HM—half-marathon. M/UM—marathon/ultra-marathon.

Figure 2 displays differences in food clusters between 10-km, HM, and M/UM runners, and further details about the regression results, including *p*-values are shown in Table 4. Assuming HM distance as the reference group, 10-km distance was a significant predicator of “fruit and vegetables” intake (i.e., FC-3) (*p* = 0.006), while M/UM distance was a significant predicator of “free/added sugar” consumption (i.e., FC-17) (*p* = 0.030). Age has been found to be a significant predictor of five food clusters, including “fruit and vegetables” (*p* = 0.003), “unprocessed meat” (*p* = 0.027), “processed meat” (*p* = 0.022), “eggs” (*p* = 0.040), and “plant protein” (*p* = 0.029).

## 4. Discussion

The present study was performed to investigate dietary intake of distance runners and to examine the potential associations between race distance (10-km, HM, M/UM) and dietary intake (assessed by FFQ, based on 14 basic food clusters and three umbrella food clusters). Results most importantly showed that (1) 10-km runners had a significant higher intake of two clusters, “fruits and vegetables” and “total of protein”, compared to runners over HM and M/UM distances; (2) no significant association was found between race distance and 15 (out of 17) food clusters; (3) “health & wellbeing” was identified as the most popular motive to follow the diet types reported in the present study and was the only motive (out of 11 motives) with a significant difference between race distance groups; (4) and race distance was found to predict the consumption of only one food cluster (“fruits and vegetables”), whereas age was found to predict five (out of 17) food clusters. To the best of the authors’ knowledge, the present study is the first investigation performed on endurance runners to examine a complete profile of their dietary intake across different race distances, including diet type subgroups.

Dietary assessment is a practical strategy to identify nutritional insufficiency helping to develop nutritional plans for optimizing performance and health in athletes and to promote health-related approaches in general populations [41,42]. For clinical and scientific purposes, the most common methods of dietary assessment include dietary records, in-depth interviews, 24-h dietary recalls, and the food frequency questionnaire (FFQ) [41,42,43]. It has been reported that food records, dietary recalls, and detailed interviews take lots of time and energy when used to investigate athletic populations [44,45]. Nevertheless, while FFQs have been found to be a simple, fast, and low-cost assessment tool with a low burden on participants [46], reports indicate that the FFQ is the most proper survey method to assess the dietary intake of athletes [46,47]. Compared to sedentary people, athletes are shown to be at a higher risk of low energy supply, and this risk increases for athletes with unbalanced or inappropriately planned diets [5,48]. Given the importance of diet and nutritional supply in health and performance, dietary assessment is the first and most crucial step in any sports nutrition practice and is necessary for personalized nutritional strategies [45]. Considering the differences in physiological and nutritional demands and challenges of runners over different distances, practical knowledge regarding their dietary habits may be helpful in designing more specified dietary plans and successive nutritional strategies.

Available literature shows that race distance is considered a significant indicator of training behaviors [22,49], which may also be linked to nutritional strategies [26]. Runners over longer distances (i.e., marathoners and ultra-marathoners) not only rely mostly on aerobic metabolism to utilize their glycogen and fat stores efficiently [50] but they also usually cope with several physical and physiological challenges, including pains during the recovery periods [20,51]. These facts may theoretically suggest their dietary and nutritional habits should differ from those of shorter-distance runners; however, general results from the present study indicate that there is no association between race distance and the consumption of most food clusters, at least independently of competition-related nutritional strategies. Consistently, it has been indicated that there is no association between race distance and the patterns of supplement intake [52] or health-related behaviors associated with food choices [4]. Findings from another study show there being no difference in diet quality between ultra-endurance runners competing over different distances [53]. Together, these findings can be partially linked to the conclusion of Thompson [54], indicating that there seems to be similar physiological demands between shorter- and longer-distance runners.

It has been reported that endurance runners generally consume more fruits and vegetables than normal populations [23]; however, to the best of the authors knowledge, there is no comparative information between shorter versus longer distance runners in patterns of fruit and vegetable intake. In the present study, a significant difference was found between race distance groups in consumption of fruits and vegetables, with the dominance of 10-km runners. Further analysis of dietary items showed that this consumption difference belongs only to vegetables (not fruits or vegetable juices). The unbalanced sex distribution of the study groups (particularly the greater number of female participants in the 10-km group) seems to justify the higher consumption of “fruit and vegetables” by 10-km runners. In this regard, female runners are found to be more interested than males in consumption of fruits and vegetables [55,56], and consistently, this food group seems to have the highest contrast in dietary patterns between males and females [57]. This might be associated with the heightened concerns over body image within female populations, specifically female athletes [58,59].

Unlike micronutrients, which can be partially synthesized in the body from different metabolic pathways, macronutrients need to be supplied completely via dietary intake [60]. While carbohydrates and fats play a central role in fueling and recovery of endurance activities [61,62], adequate protein intake is crucial for maintaining muscle protein synthesis, optimizing recovery processes, regulating immune function, and balancing hormonal and enzymatic activities of endurance runners [63]. In the present study, there was no difference between race distance groups in consumption of carbohydrate-, fat-, and protein-based food items. However, analysis of umbrella food clusters showed a significant between-group difference, where 10-km runners reported a higher consumption of “total protein”. Results from a study show that endurance runners seem to consume healthier carbohydrate and protein foods than normal populations, with a higher consumption of unrefined grains, white meats, and low-fat dairy products, and a lower consumption of refined grains, red meat, and high-fat dairy products [23]. Inconsistently, a lower consumption of whole grains has also been reported in endurance runners compared to non-athletes [64]. Another study showed that distance runners had a lower consumption of carbohydrates compared to other endurance athletes [65]. Differentiated findings from an investigation on ultra-marathoners indicate that there is a wide variation in their dietary intake, but it appears that they generally follow low-carbohydrate (high-protein and high-fat) diets compared to the dietary recommendations for endurance athletes [66]. While it has been well-established that training/racing behaviors of endurance runners are associated with their carbohydrate intake [23,61], dietary recommendations emphasize that endurance runners should generally consume higher amounts of carbohydrates compared to general populations [67,68]. A higher percentage of daily carbohydrates fulfill the required energy for performance and recovery of ultra-endurance activities [18]. Beyond these basic facts, it is recommended that carbohydrate intake should be based on sport-specific needs, training features, and individual differences in nutritional and physiological characteristics [69,70]. However, it is necessary to consider dietary intake assessed by analysis of food clusters is different than nutritional evaluation based on calorie assessment, and thus, caution is advised in interpreting nutritional aspects and the associated representativeness in endurance athletes.

In this present study, findings on hydration habits showed that race distance is not an influencing factor in the consumption of water, beverages, or alcohol. Analysis of dietary items also showed no difference between race distance groups in vegetable juice and milk intake. Research indicated that training and racing behaviors of endurance runners play a stronger role in their hydration-related dietary patterns [71]. Evidence shows that endurance runners (especially female runners) have a lower amount and frequency of alcohol intake compared to general populations [72]. Results from another investigation comparing a large number of endurance runners with non-athletes show that runners reported drinking more water but less coffee, sweet drinks, and alcoholic beverages, yet there was no difference between the total daily fluid intake [23]. In this regard, it has been found that the majority of marathoners rely on a self-structured plan for fluid intake, as they are confident in their ability to hydrate [25]. In general, caution should be considered when making a conclusion about endurance runner fluid intake since training characteristics (e.g., duration and intensity) and environmental variables (e.g., temperature and humidity) potentially influence hydration balance.

Although available literature indicates that endurance runners rely more on self-planned dietary strategies in preparation and competition phases [73], distance runners generally have acceptable health-related behaviors for food choice and dietary ingredients [4,74]. In this present study, differences between 10-km, HM, and M/UM runners in the consumption of 17 dietary clusters were not sufficient to recognize any health-related tendency in their diet. While the higher level of health consciousness in endurance runners compared to general populations may partially explain this finding [4,75], it should be considered that participants in the present study were predominately recreational runners who may have different choices of dietary intake compared to professional runners, which originates from their dissimilar goals for engaging in training and competition [76]. A minor tendency towards a better health status has been reported in half-marathon runners compared to runners competing over shorter and longer distances [4]. Inconsistently, a recent study found no difference in the quality of diets between ultra-endurance versus other runners [74]. This is not in line with a finding from Heikura et al. [21], indicating that training under a fasting state is two-times more prevalent in ultra-endurance runners than endurance runners, which is probably due to weight reduction and/or optimizing the training adaptations.

In line with the present study in which a considerable portion of runners were vegans and vegetarians, data indicate that the popularity of vegan and vegetarian diets in endurance runners is higher than general populations [23,66,73]. Research shows that 10% of distance runners follow a vegan/vegetarian diet, with a higher prevalence in runners competing over longer distances such as ultramarathon [25,53]. Runners who adhere to plant-based diets are reported to have a higher score of diet quality compared to their omnivorous peers [53]. Consistently, distance runners are found to consume more plant-based proteins than animal proteins [77], while dietary protein of athletic populations seems to be mostly derived from animal sources [78]. Data have shown that the consumption of more plant-based foods and less animal foods may independently result in a lower consumption of processed and high-fat foods, including fast foods [79], increasing the overall nutritional value of the diet [80]. It has been previously reported that endurance runners who follow plant-based diets have greater health consciousness compared to omnivorous runners [28]. Regardless of diet type, educational level (specifically having basic knowledge on sport nutrition practices) may also be associated with advanced health behaviors in distance runners, particularly in choosing healthier dietary strategies [3]. However, in terms of academic qualification, no significant difference was found between 10-km, HM, and M/UM runners in the present study. In this study, age was a significant predictor for consumption of five food groups, including “fruit and vegetables”, “unprocessed meat”, “processed meat”, “eggs”, and “plant protein”. Available data from dietary studies demonstrate that age is considered a moderate indicator of dietary behaviors in general populations [57,81]. In studies on athletic populations, age can also be linked to the term “professionalism”, which is known to be a significant indicator of dietary strategy associated with training and racing requirements [82,83]. Consistently, this might be in line with the present age-related findings in which most participants were recreational runners.

There are some limitations necessary to notify. The cross-sectional design using self-reported data might result in under- and/or over-reporting, which seems to be prevalent in investigations on athletes [41]. However, a variety of control questions were designed and implemented in the questionnaires to recognize and revise contradictory data in order to minimize the bias level. On this point, raw data were clearly checked for congruency and meaningfulness. In the present study, the sex-based heterogeneity among study groups may be considered another limitation influencing the dietary findings and the associated interpretations, since females, who were more distributed in shorter distances, are well-known to be generally more health-conscious than males [54,84]. Although FFQ is a well-approved method to evaluate dietary intake [45,46], and especially in athletic populations [46,47], this method may not fully provide data about the micro- and macro-nutrient status (which most dietary guidelines for athletes are based on). However, it should be considered that dietary guidelines are usually based on conventional definitions of the food groups and do not consider the food processing aspects, which may not be practically applicable [85].

Despite the aforementioned facts, the present results can be considered novel data that add to current scientific knowledge about the association of race distance with dietary intake in endurance runners. The findings may provide new directions for future studies, including clinical trials focusing on athletic populations. Differentiated results based on race distance provide a new window into the targeted approaches aiming to develop specified nutritional strategies matched with the well-approved physiological and behavioral differences in training and competition of runners over shorter vs. longer distances. Such studies provide a firm knowledge helping to design and apply nutritional strategies to optimize health and performance. Endurance runners and their coaches, sports dietitians, and sports nutrition specialists can use these results when designing and applying the most appropriate and personalized nutritional strategies.

## 5. Conclusions

The popularity of distance running is growing worldwide but endurance runners’ dietary challenges, which originate from their prolonged training and racing activities, have not yet been fully addressed. The present examination of distance runners’ dietary intake assessed by food frequency questionnaire revealed that except for two food clusters (i.e., “fruits and vegetables” and “total protein”), there are mostly insignificant differences in food choices between 10-km, half-marathon, and (ultra-)marathon runners. Despite some previous data indicating physiological and health-related behavioral differences between runners competing over different distances, the present findings suggest that dietary patterns of 10 km, HM, and M/UM runners are not influenced by such differences. Findings from the present study along with those derived from previous investigations (including the NURMI Study) may indicate that there are more potential variables (e.g., sex, age, diet type, and training behaviors) than race distance affecting dietary patterns. Future investigations on endurance runners with larger samples in differentiated subgroups based on race distance (5 km, 10 km, HM, M, and UM) as well as sociodemographic characteristics (age groups, BMI categories, professional vs. recreational runners, etc.) are necessary to help make comparable data and deliver a more advanced understanding of the dietary patterns among endurance runners. However, the present findings may contribute in helping provide more precise, race-distance related dietary approaches, aiding to develop personalized strategies in sports nutrition practice and counseling tailored to specific race distances.

## Figures and Tables

**Table 1:** Modeling of food items, groups, and clusters (including 14 basic consumption clusters along with 3 umbrella/preparation clusters.

**Basic Food Clusters**		
Cluster 1	Grains	
	a-grains	white bread; white pasta; cornflakes
	b-whole grains	wholegrain; mixed bread; muesli; wholegrain pasta; wholegrain rice; other grains
Cluster 2	Legumes, nuts, and pulses	pulses; legumes; nuts and seeds
Cluster 3	Fruit and vegetables	vegetable juice; vegetables; fruit
Cluster 4	Dairy products	cheese; milk; yogurt
Cluster 5	Dairy alternatives	milk alternatives
Cluster 6	Meat	
	a-meat	beef; chicken; pork; deer
	b-processed meat	hamburger; sausage; fried nuggets; kebab; pork; processed meat
Cluster 7	Meat alternatives	tofu; tempeh; seitan; etc.
Cluster 8	Fish, shellfish, and seafood	
Cluster 9	Eggs	
Cluster 10	Oils and spreads	butter; margarine; oils
Cluster 11	Sweets and snacks	snacks; sweets; salty snacks
Cluster 12	Water and unsweetened tea	
Cluster 13	Beverages	
Cluster 14	Alcohol	
**Preparation/Umbrella Clusters**		
Cluster 15	Protein	
	a-plant protein	legumes and beans; grains (couscous, quinoa); vegetables; dairy alternatives (e.g., soy products); meat alternatives
	b-animal protein	eggs; meat and processed meat products; dairy products; fish, seafood, and shellfish
Cluster 16	(Ultra-)processed foods and free/added sugar	kcal reduced/artificially sweetened drinks; sugary carbonated drinks; fruit juice; free sugar in tea; free sugar in coffee; cereals; sweet and savory spreads; pasta; sweets, cakes, and biscuits; salty snacks, margarine; butter; processed meat; processed plant products
Cluster 17	Free/added sugar	sugary carbonated drinks; fruit juice; free sugar in tea; free sugar in coffee; sweet spread; cereals; sweets, cakes, and biscuits

**Table 2:** Sociodemographic characteristics of the runners across race distance groups.

		Total *n* = 211	10-km *n* = 74	HM *n* = 83	M/UM *n* = 54	Statistics
Age (years)		38 (IQR 18)	36 (IQR 17)	37 (IQR 18)	43 (IQR 16)	F_(2, 208)_ = 2.89; *p* = 0.058
Sex	Females	57% (121)	74% (55)	55% (46)	37% (20)	χ^2^_(2)_ = 17.95; *p* < 0.001
	Males	43% (90)	26% (19)	45% (37)	63% (34)	
Body weight (kg)		65.0 (IQR 14.1)	62.1 (IQR 11.7)	65.0 (IQR 12.3)	69.3 (IQR 18.4)	F_(2, 208)_ = 5.85; *p* = 0.003
Height (m)		1.7 (IQR 0.1)	1.7 (IQR 0.1)	1.7 (IQR 0.1)	1.8 (IQR 0.1)	F_(2, 208)_ = 5.71; *p* = 0.004
BMI (kg/m2)		21.7 (IQR 3.4)	21.3 (IQR 3.9)	21.9 (IQR 3.4)	22.2 (IQR 2.9)	F_(2, 208)_ = 1.78; *p* = 0.172
Marital Status	Divorced/Separated	5% (11)	5% (4)	5% (4)	6% (3)	χ^2^_(4)_ = 1.05; *p* = 0.903
	Married/Partner	68% (143)	68% (50)	65% (54)	72% (39)	
	Single	27% (57)	27% (20)	30% (25)	22% (12)	
Academic Qualification	Upper Secondary	33% (69)	23% (17)	37% (31)	39% (21)	χ^2^_(6)_ = 13.40; *p* = 0.099
	A Level or Equivalent	23% (49)	30% (22)	17% (14)	24% (13)	
	University/College	34% (72)	42% (31)	30% (25)	30% (16)	
	No Answer	9% (21)	5% (4)	15% (13)	7% (4)	
Nationality	Austria	17% (36)	18% (13)	17% (14)	17% (9)	χ^2^_(6)_ = 2.47; *p* = 0.871
	Germany	74% (156)	74% (55)	75% (62)	72% (39)	
	Switzerland	5% (11)	3% (2)	6% (5)	7% (4)	
	Other Countries	4% (8)	5% (4)	2% (2)	4% (2)	
Diet Type	Omnivorous	45% (95)	46% (34)	43% (36)	37% (20)	χ^2^_(4)_ = 1.41; *p* = 0.843
	Vegetarian	19% (40)	16% (12)	23% (19)	17% (9)	
	Vegan	36% (76)	38% (28)	34% (28)	37% (20)	
Motives for Specified Diet Type	Health & Wellbeing	85% (106)	74% (32)	94% (49)	86% (25)	χ^2^_(2)_ = 7.46; *p* = 0.024
	Sport Performance	51% (63)	42% (18)	54% (28)	59% (17)	χ^2^_(2)_ = 2.28; *p* = 0.320
	Food Scandals	35% (44)	33% (14)	37% (19)	38% (11)	χ^2^_(2)_ = 0.26; *p* = 0.877
	Animal Ethics	78% (97)	77% (33)	79% (41)	79% (23)	χ^2^_(2)_ = 0.09; *p* = 0.957
	Ecological Aspects	73% (91)	74% (32)	65% (34)	86% (25)	χ^2^_(2)_ = 4.17; *p* = 0.124
	Social Aspects	49% (61)	63% (27)	42% (22)	41% (12)	χ^2^_(2)_ = 4.88; *p* = 0.087
	Economic Aspects	18% (22)	21% (9)	17% (9)	14% (4)	χ^2^_(2)_ = 0.62; *p* = 0.735
	Religion/ Spirituality	6% (8)	7% (3)	6% (3)	7% (2)	χ^2^_(2)_ = 0.07; *p* = 0.966
	Custom/Tradition	5% (6)	7% (3)	4% (2)	3% (1)	χ^2^_(2)_ = 0.66 *p* = 0.719
	Taste & Enjoyment	44% (54)	37% (16)	44% (23)	52% (15)	χ^2^_(2)_ = 1.50; *p* = 0.472
	No Specified Reason	<1% (1)	-	2% (1)	-	χ^2^_(2)_ = 1.40; *p* = 0.498

Note. Data are presented as median (IQR) or percentage (*n*). IQR—Interquartile range. BMI—body mass index. km—kilometers. HM—half-marathon. M/UM—marathon/ultra-marathon. Statistical methods: Kruskal–Wallis tests (F-values) and Chi-square tests (χ^2^).

**Table 3:** Differences between 10-km, HM, and M/UM runners in food frequency clusters and items.

	10-km *n* = 74	HM *n* = 83	M/UM *n* = 54	Statistics
**Part A—Basic Clusters**				
FC—1: Total of grains	17.41 ± 7.75	18.35 ± 7.85	19.00 ± 10.64	F_(2, 208)_ = 0.49; *p* = 0.610
FC—1a (Total of refined grains)	12.02 ± 9.06	12.81 ± 8.75	12.12 ± 10.10	F_(2, 208)_ = 0.43; *p* = 0.651
Cornflakes	2.41 ± 5.65	1.13 ± 3.23	0.96 ± 3.00	F_(2, 208)_ = 2.77; *p* = 0.065
White bread	6.92 ± 7.26	8.31 ± 8.24	8.59 ± 8.40	F_(2, 208)_ = 0.64; *p* = 0.530
White pasta	10.51 ± 9.86	11.69 ± 7.90	10.44 ± 10.22	F_(2, 208)_ = 1.10; *p* = 0.333
FC—1b (Total of whole grains)	18.57 ± 8.31	19.45 ± 8.36	21.28 ± 11.38	F_(2, 208)_ = 1.26; *p* = 0.286
Muesli	15.15 ± 12.86	16.00 ± 12.66	19.28 ± 14.16	F_(2, 206)_ = 1.44; *p* = 0.239
Whole grain bread	14.89 ± 8.18	17.41 ± 9.02	16.87 ± 10.52	F_(2, 208)_ = 1.73; *p* = 0.180
Whole grain pasta	10.76 ± 9.17	9.52 ± 7.44	10.33 ± 9.82	F_(2, 208)_ = 0.22; *p* = 0.804
Whole grain rice	7.43 ± 7.99	6.29 ± 6.81	8.22 ± 7.72	F_(2, 208)_ = 1.01; *p* = 0.368
Other whole grains	6.92 ± 7.26	8.31 ± 8.24	8.59 ± 8.40	F_(2, 208)_ = 0.64; *p* = 0.530
FC—2: Total of beans and seeds	28.40 ± 14.14	24.96 ± 14.22	26.01 ± 13.37	F_(2, 208)_ = 1.48; *p* = 0.229
Nuts & seeds	21.81 ± 13.54	18.84 ± 13.68	17.85 ± 12.19	F_(2, 208)_ = 1.87; *p* = 0.157
Legumes	16.32 ± 11.12	14.67 ± 9.27	17.07 ± 11.97	F_(2, 208)_ = 0.52; *p* = 0.596
FC—3: Total of fruit and vegetables	34.73 ± 13.60	28.98 ± 12.23	28.97 ± 12.33	F_(2, 208)_ = 4.68; *p* = 0.010
Vegetable juice	6.70 ± 11.08	3.90 ± 8.18	6.59 ± 12.66	F_(2, 208)_ = 0.82; *p* = 0.440
Fruit	18.97 ± 10.24	18.71 ± 7.69	20.17 ± 9.45	F_(2, 208)_ = 0.37; *p* = 0.689
Vegetables	34.78 ± 12.99	30.20 ± 11.80	28.85 ± 11.27	F_(2, 208)_ = 4.53; *p* = 0.012
FC—4: Total of dairy	10.86 ± 12.16	8.74 ± 10.10	11.38 ± 11.11	F_(2, 208)_ = 0.79; *p* = 0.455
Milk	8.65 ± 10.66	8.43 ± 12.45	8.26 ± 11.28	F_(2, 208)_ = 0.15; *p* = 0.859
Cheese	7.91 ± 8.84	6.35 ± 7.74	8.83 ± 9.18	F_(2, 208)_ = 1.05; *p* = 0.353
Yogurt	8.61 ± 11.66	5.46 ± 7.76	9.26 ± 11.02	F_(2, 208)_ = 1.69; *p* = 0.186
FC—5: Dairy alternatives	16.11 ± 14.71	16.02 ± 15.64	16.63 ± 16.07	F_(2, 208)_ = 0.03; *p* = 0.969
FC—6: Total of meat	7.41 ± 11.56	8.72 ± 13.08	8.30 ± 11.75	F_(2, 208)_ = 0.27; *p* = 0.761
FC—6a (Total of unprocessed meat)	8.05 ± 12.52	9.01 ± 13.67	9.01 ± 12.67	F_(2, 208)_ = 0.21; *p* = 0.808
Chicken	3.71 ± 6.07	3.23 ± 5.60	3.67 ± 5.90	F_(2, 208)_ = 0.07; *p* = 0.935
Beef, pork, or deer	6.32 ± 10.54	7.98 ± 12.34	7.55 ± 11.31	F_(2, 208)_ = 0.27; *p* = 0.762
FC—6b (Total of processed meat)	5.95 ± 10.14	7.49 ± 11.88	6.68 ± 10.48	F_(2, 208)_ = 0.17; *p* = 0.843
Fried nuggets	1.97 ± 3.30	1.76 ± 3.66	1.93 ± 3.34	F_(2, 208)_ = 0.11; *p* = 0.893
Hamburger	0.78 ± 2.22	0.99 ± 2.24	1.15 ± 2.79	F_(2, 208)_ = 0.32; *p* = 0.727
Sausage	0.51 ± 1.93	0.99 ± 2.68	0.78 ± 2.21	F_(2, 208)_ = 0.82; *p* = 0.441
Kebab	0.65 ± 1.88	0.99 ± 2.13	0.96 ± 2.20	F_(2, 208)_ = 0.82; *p* = 0.442
Other processed meat	5.76 ± 11.07	7.45 ± 12.89	6.04 ± 9.65	F_(2, 208)_ = 0.24; *p* = 0.784
FC—7: Meat alternatives	6.17 ± 6.64	5.75 ± 6.46	6.38 ± 7.05	F_(2, 208)_ = 0.21; *p* = 0.807
FC—8: Fish	5.30 ± 6.82	4.29 ± 6.02	3.94 ± 5.94	F_(2, 208)_ = 0.55; *p* = 0.580
FC—9: Eggs	8.00 ± 8.70	7.11 ± 8.67	8.85 ± 9.15	F_(2, 208)_ = 0.75; *p* = 0.473
FC—10: Total of oils	11.65 ± 11.14	12.76 ± 13.18	13.19 ± 14.83	F_(2, 208)_ = 0.03; *p* = 0.969
Butter	5.49 ± 9.54	5.45 ± 11.03	7.52 ± 13.28	F_(2, 208)_ = 0.13; *p* = 0.877
Margarine	6.30 ± 9.62	7.35 ± 10.59	5.81 ± 9.45	F_(2, 208)_ = 0.66; *p* = 0.516
Other oils	5.69 ± 5.59	6.35 ± 6.62	6.44 ± 7.47	F_(2, 208)_ = 0.08; *p* = 0.919
FC—11: Total of snacks	11.42 ± 7.34	10.48 ± 6.81	9.97 ± 7.40	F_(2, 208)_ = 0.77; *p* = 0.464
Sweet snacks	10.29 ± 6.09	10.07 ± 6.84	9.83 ± 6.91	F_(2, 208)_ = 0.27; *p* = 0.761
Salty snacks	7.21 ± 7.80	5.90 ± 7.14	5.47 ± 6.29	F_(2, 206)_ = 0.77; *p* = 0.466
FC—12: Total of water	34.08 ± 19.93	36.20 ± 21.41	35.54 ± 22.10	F_(2, 208)_ = 0.08; *p* = 0.925
Water	56.24 ± 26.31	61.40 ± 28.42	60.89 ± 28.08	F_(2, 208)_ = 0.92; *p* = 0.400
Unsweetened tea	22.92 ± 14.76	21.07 ± 17.73	20.56 ± 18.18	F_(2, 208)_ = 1.79; *p* = 0.170
FC—13: Beverages	13.60 ± 4.87	13.81 ± 5.00	14.27 ± 5.08	F_(2, 208)_ = 0.33; *p* = 0.718
FC—14: Alcohol	3.73 ± 4.76	3.90 ± 5.15	3.50 ± 4.29	F_(2, 208)_ = 0.09; *p* = 0.914
**Part B—Umbrella Clusters**				
FC—15: Total of protein	42.40 ± 13.82	36.14 ± 14.21	39.49 ± 13.42	F_(2, 208)_ = 4.24; *p* = 0.016
15a (Plant protein)	35.73 ± 14.52	31.35 ± 14.99	31.98 ± 14.13	F_(2, 208)_ = 2.38; *p* = 0.095
FC—15b (Animal protein)	15.67 ± 15.35	14.40 ± 15.13	16.29 ± 14.85	F_(2, 208)_ = 0.20; *p* = 0.822
FC—16: Processed foods & free/added sugar	25.05 ± 12.64	28.13 ± 15.47	25.00 ± 14.79	F_(2, 208)_ = 1.34; *p* = 0.264
FC—17: Free/added sugar	13.98 ± 9.33	16.57 ± 10.83	12.86 ± 8.60	F_(2, 208)_ = 2.23; *p* = 0.110

Note. Data are presented as Mean ± Standard Deviation. FC—food clusters. Km—kilometers. HM—half-marathon. M/UM—marathon/ultra-marathon. Statistical method: Kruskal–Wallis tests (F-values).

**Table 4:** Linear regression results for age- and race distance-related main effects on food clusters with HM runners as the reference group.

	Age			HM vs. 10-km			HM vs. M/UM		
	*β*	95%-CI	*p*	*β*	95%-CI	*p*	*β*	95%-CI	*p*
**Part A—Basic Clusters**									
FC—1a: Total of refined grains	0.42	[1.62, −0.78]	0.495	−0.76	[2.15, −3.67]	0.608	−0.85	[2.37, −4.06]	0.603
FC—1b: Total of whole grains	−0.17	[1.03, −1.36]	0.786	−0.88	[2.03, −3.79]	0.550	1.90	[5.11, −1.32]	0.246
FC—2: Total of beans and seeds	−0.71	[1.11, −2.53]	0.444	3.39	[7.81, −1.02]	0.131	1.33	[6.20, −3.55]	0.593
FC—3: Total of fruit and vegetables	−2.46	[−0.83, −4.09]	0.003	5.58	[9.53, 1.64]	0.006	0.96	[5.32, −3.40]	0.666
FC—4: Total of dairy	0.42	[1.87, −1.02]	0.563	2.15	[5.66, −1.36]	0.228	2.47	[6.35, −1.41]	0.211
FC—5: Dairy alternatives	−0.45	[1.56, −2.46]	0.661	0.05	[4.93, −4.82]	0.982	0.78	[6.17, −4.61]	0.775
FC—6a: Total of unprocessed meat	1.89	[3.57, 0.21]	0.027	−0.83	[3.24, −4.90]	0.688	−0.74	[3.76, −5.23]	0.747
FC—6b: Total of processed meat	1.65	[3.05, 0.24]	0.022	−1.43	[1.99, −4.84]	0.411	−1.46	[2.32, −5.23]	0.448
FC—7: Meat alternatives	0.31	[1.17, −0.56]	0.489	0.44	[2.55, −1.67]	0.681	0.51	[2.84, −1.82]	0.665
FC—8: Fish	0.71	[1.52, −0.11]	0.089	1.05	[3.03, −0.92]	0.294	−0.62	[1.56, −2.80]	0.575
FC—9: Eggs	1.19	[2.33, 0.06]	0.040	0.97	[3.72, −1.79]	0.489	1.28	[4.32, −1.77]	0.410
FC—10: Total of oils	1.23	[2.91, −0.45]	0.150	−1.03	[3.04, −5.11]	0.618	−0.06	[4.45, −4.57]	0.979
FC—11: Total of snacks	0.13	[1.07, −0.80]	0.778	0.95	[3.21, −1.31]	0.410	−0.56	[1.94, −3.06]	0.659
FC—12: Total of water	−2.09	[0.64, −4.83]	0.133	−2.26	[4.37, −8.89]	0.503	0.16	[7.49, −7.16]	0.965
FC—13: Beverages	0.19	[0.83, −0.46]	0.573	−0.20	[1.38, −1.77]	0.807	0.39	[2.13, −1.35]	0.659
FC—14: Alcohol	0.33	[0.96, −0.29]	0.293	−0.15	[1.37, −1.66]	0.847	−0.53	[1.15, −2.20]	0.535
**Part B—Umbrella Clusters**									
FC—15a: Plant protein	−2.10	[−0.22, −3.98]	0.029	4.24	[8.80, −0.33]	0.069	1.45	[6.50, −3.59]	0.571
FC—15b: Animal protein	1.90	[3.86, −0.06]	0.057	1.40	[6.14, −3.34]	0.561	1.15	[6.39, −4.09]	0.667
FC—16: Processed foods & free/added sugar	0.53	[2.40, −1.34]	0.578	−3.04	[1.49, −7.58]	0.187	−3.33	[1.68, −8.34]	0.191
FC—17: Free/added sugar	0.23	[1.50, −1.05]	0.724	−2.57	[0.52, −5.66]	0.102	−3.80	[−0.38, −7.21]	0.030

Note. HM—half-marathon. M/UM—marathon/ultra-marathon. *Β*—standardized regression coefficient. CI—confidence interval. *p*—*p*-value.

## Data Availability

The datasets generated and/or analyzed during different steps of the study are not publicly available but may be made available upon reasonable request. Participants will receive a brief summary of the findings of the “NURMI Study” upon request.
